# Mitochondrial genome complexity in *Stemona sessilifolia*: nanopore sequencing reveals chloroplast gene transfer and DNA rearrangements

**DOI:** 10.3389/fgene.2024.1395805

**Published:** 2024-06-04

**Authors:** Yuning Xie, Wenqiong Liu, Liwen Guo, Xuemei Zhang

**Affiliations:** ^1^ School of Public Health, North China University of Science and Technology, Tangshan, China; ^2^ Public Health Department, Affiliated Hospital of Shandong University of Traditional Chinese Medicine, Jinan, China; ^3^ College of Life Science, North China University of Science and Technology, Tangshan, China

**Keywords:** *Stemona sessilifolia*, mitochondrial genome, repeated sequences, phylogenetic relationship, RNA editing

## Abstract

Mitochondria are semi-autonomous organelles in eukaryotic cells with their own genome. Plant mitogenomes differ from animal mitogenomes in size, structure, and repetitive DNA sequences. Despite larger sizes, plant mitogenomes do not have significantly more genes. They exhibit diverse structures due to variations in size, repetitive DNA, recombination frequencies, low gene densities, and reduced nucleotide substitution rates. In this study, we analyzed the mitochondrial genome of *Stemona sessilifolia* using Nanopore and Illumina sequencing. *De-novo* assembly and annotation were conducted using Unicycler, Geseq, tRNAscan-SE and BLASTN, followed by codon usage, repeat sequence, RNA-editing, synteny, and phylogenetic analyses. *S. sessilifolia’s* mitogenome consisted of one linear contig and six circular contigs totaling 724,751 bp. It had 39 protein-coding genes, 27 tRNA genes, and 3 rRNA genes. Transfer of chloroplast sequences accounted for 13.14% of the mitogenome. Various analyses provided insights into genetic characteristics, evolutionary dynamics, and phylogenetic placement. Further investigations can explore transferred genes’ functions and RNA-editing’s role in mitochondrial gene expression in *S. sessilifolia*.

## 1 Introduction

Mitochondria are widely distributed semi-autonomous organelles within eukaryotic cells that harbor their own genome, known as the mitochondrial genome which consists of circular double-helix DNA (Saccone et al., 2000). Mitochondrial genome may also comprise other small circular chromosomes or sub-genomes (Wanrooij et al., 2012; Sloan, 2013). Previous study illustrated mitogenomes using circular maps, suggesting their physical presence as circular monomeric DNA molecules (Manchekar et al., 2006); however, researchers also demonstrated that the physical structure of mitochondrial genomes is not exclusively confined to a circular monomeric form (Menger et al., 2021). For instance, to examine the mitogenomic structure of by fluorescence microscopy technique, Kozik and his colleagues discovered that the mitochondrial genome of Lactuca sativa exhibited linear, branched, and circular configurations (Kozik et al., 2019).

Plant mitogenomes are larger and more complex in nature in comparison to animal mitogenomes, which typically span around 15–17 kb in length. Plant mitogenomes vary in size, ranging from 200 kb to 11 Mb in length, but do not harbor much more genes (Zardoya, 2020). The structure of mitochondrial genomes (mitogenomes) in flowering plants displays remarkable diversity, including variations in size, repetitive DNA sequences, recombination frequencies of extensive repeats, low gene densities, and reduced rates of nucleotide substitution (Rose, 2021; Maliga, 2022). The expansion of plant mitogenomes primarily stems from the accumulation of repetitive sequences and the incorporation of DNA derived from chloroplast and nuclear genomes (Petit et al., 2005; Greiner and Bock, 2013; Li et al., 2016; Zhang et al., 2020b; Tan et al., 2022). Consequently, these intricate genomes pose challenges for assembly using short paired-end read sequencing.

*Stemona sessilifolia (Miq.) Miq.*, commonly known as Zhili-Baibu, is a highly esteemed and extensively used in traditional Chinese medicine (Chanmahasathien et al., 2011). Stemonae Radix, derived from the roots of three species: *S. sessilifolia* (Yang et al., 2009), *S. japonica* (Tan et al., 2023), and S*. tuberosa* (Fan et al., 2015), has been widely used for a considerable time due to its antitussive and insecticidal properties. For *S. sessilifolia*, the nuclear and chloroplast genomes of have not been thoroughly annotated, and its mitogenome remains unexamined.

In this study, we present the first assembled and annotated mitogenome of *S. sessilifolia*, employing a combination of Nanopore and Illumina sequencing technologies with *S. sessilifolia* as the genetic material. These findings offer further substantiation for the existence of diverse conformations within plant mitogenomes, while also providing valuable insights for future investigations into the phylogenetic status of *S. sessilifolia*.

## 2 Materials and methods

### 2.1 Materials and sequencing

In June 2023, fresh leaves of *S. sessilifolia* were collected from Moyun Mountain, located in Jinan City, Shandong Province (coordinates: N 36°20′31.0308″, E 117°54′43.4772″). *S. sessilifolia* is not classified as an endangered or protected species, thus no specific permission was required for the collection. All samples were thoroughly rinsed, cleaned using DEPC water, and subsequently stored at −80°C. Total DNA was extracted using TIANamp Genomic DNA Kit (Tiangen, Beijing, China). To obtain comprehensive data, we sequenced the mitogenome of *S. sessilifolia* on both Nanopore GridION sequencing platform (Oxford Nanopore Technology, Oxford Science Park) and Illumina Novaseq 6000 platform (Illumina, San Diego, USA), which enabling the construction of libraries and the generation of raw data (Nanopore raw data: 23.69 Gb, Illumina raw data: 25.94 Gb). The data reported in this paper have been deposited in the GenBank of NCBI (Benson et al., 2013), under accession number PP692484, PP692485, PP692486, PP692487, PP692488, PP692489 and PP692490 that were publicly accessible at https://www.ncbi.nlm.nih.gov/genbank/.

### 2.2 Assembly and annotation of organelle genomes

The *S. sessilifolia* mitogenome was assembled using a comprehensive approach combining Illumina and Nanopore sequencing technologies. Initially, we employed Flye (Kolmogorov et al., 2019) software to conduct *de novo* assembly of long reads derived from *S. sessilifolia* obtained through Oxford Nanopore sequencing. Subsequently, the BLASTn (Chen et al., 2015) was utilized to identify the draft mitogenome of *S. sessilifolia* by comparing the assembled contigs. To facilitate this process, we created a database for the assembled sequences using makeblastdb and chose conserved mitochondrial genes from *Arabidopsis thaliana* (L.) Heynh. as our query sequence to pinpoint contigs that contain these conserved mitochondrial genes. The commonly parameters used for this assembly included '-evalue 1e-5 -outfmt 6 -max_hsps 10 -word_size 7 -task blastn-short’. Additionally, we conducted a hybrid assembly using Unicycler, intergrating both Illumina short reads and Nanopore long reads (Wick et al., 2017), while applying the default parameters. For the annotation of protein-coding genes (PCGs) in the mitogenome, we selected *A. thaliana* (NC_037304) and *Liriodendron tulipifera* (NC_021152.1) as reference genomes, using Geseq for the annotation process (Tillich et al., 2017). Annotation of tRNA and rRNA within the mitogenome was accomplished using tRNAscan-SE (Chan et al., 2021) and BLASTn (Chen et al., 2015), respectively. Manual correction of annotation errors in the mitogenome was performed using Apollo (Lewis et al., 2002).

### 2.3 Analysis of codon usage and repeated sequences

Protein-coding gene (PCG) sequences were extracted from the genome using Phylosuite (Zhang et al., 2020a), and the codon usage in mitochondrial PCGs was analyzed using Mega 7.0 (Kumar et al., 2016) and relative synonymous codon usage (RSCU) values were also calculated. To identify repeated sequences, including simple sequence repeats (SSRs), tandem repeats, and interspersed repeats, MISA (Beier et al., 2017) (https://webblast.ipk-gatersleben.de/misa/), TRF (Benson, 1999) (https://tandem.bu.edu/trf/trf.unix.help.html), and REPuter (Kurtz et al., 2001) (https://bibiserv.cebitec.uni-bielefeld.de/reputer/) were employed. The results were visualized using the RCircos (Zhang et al., 2013) package.

### 2.4 Prediction of RNA editing sites

Deepred-mt (Edera et al., 2021), a tool based on the convolutional neural network (CNN) model, was utilized for predicting C to U RNA editing sites. Mitochondrial protein-coding genes were extracted for prediction analysis, and only results with probability values exceeding 0.9 were selected for further consideration.

### 2.5 Chloroplast to mitochondrion DNA transformation

The chloroplast genome was assembled and annotated using GetOrganelle (Jin et al., 2020), and CPGAVAS2, respectively (Shi et al., 2019). The BLASTn (Chen et al., 2015) program was utilized to compare two organelle genomes of S. sessilifolia. In this process, the mitogenome was established as the database with makeblastdb, and the chloroplast genome was employed as the query sequence. All results were visualized using the Circos (Krzywinski et al., 2009) package.

### 2.6 Phylogenetic inference

Related species of *S. sessilifolia* were selected based on their genetic relationship, and their complete mitogenome sequences were downloaded from NCBI (https://www.ncbi.nlm.nih.gov) (Supplementary Table S3). PhyloSuite (Zhang et al., 2020a) was utilized to extract shared mitochondrial genes across these species. Multiple sequences alignment was carried out using MAFFT (Katoh et al., 2002; Katoh et al., 2019) with a bootstrap value of 1000. IQ-TREE (Minh et al., 2020) was used for phylogenetic analysis. The resulting phylogenetic analysis was visualized using iTOL (Letunic and Bork, 2021).

### 2.7 Synteny analysis

Using the BLASTn, we identified conserved homologous sequences, which are referred to as co-linear blocks, with commonly parameters '-value 1e-5, -word_size 9, -gapopen 5, -gapextend 2, -reward 2, -penalty -3' (Shan et al., 2023). As a result, only co-linear blocks longer than 500 bp were considered. Based on sequence similarity. The mitochondrial genome of *S. sessilifolia* was compared with multiple synteny regions from closely related species using MCscanX (Stotter et al., 1990).

## 3 Results

### 3.1 Characteristics of the mitochondrial genomes of *S. sessilifolia*

**FIGURE 1:**
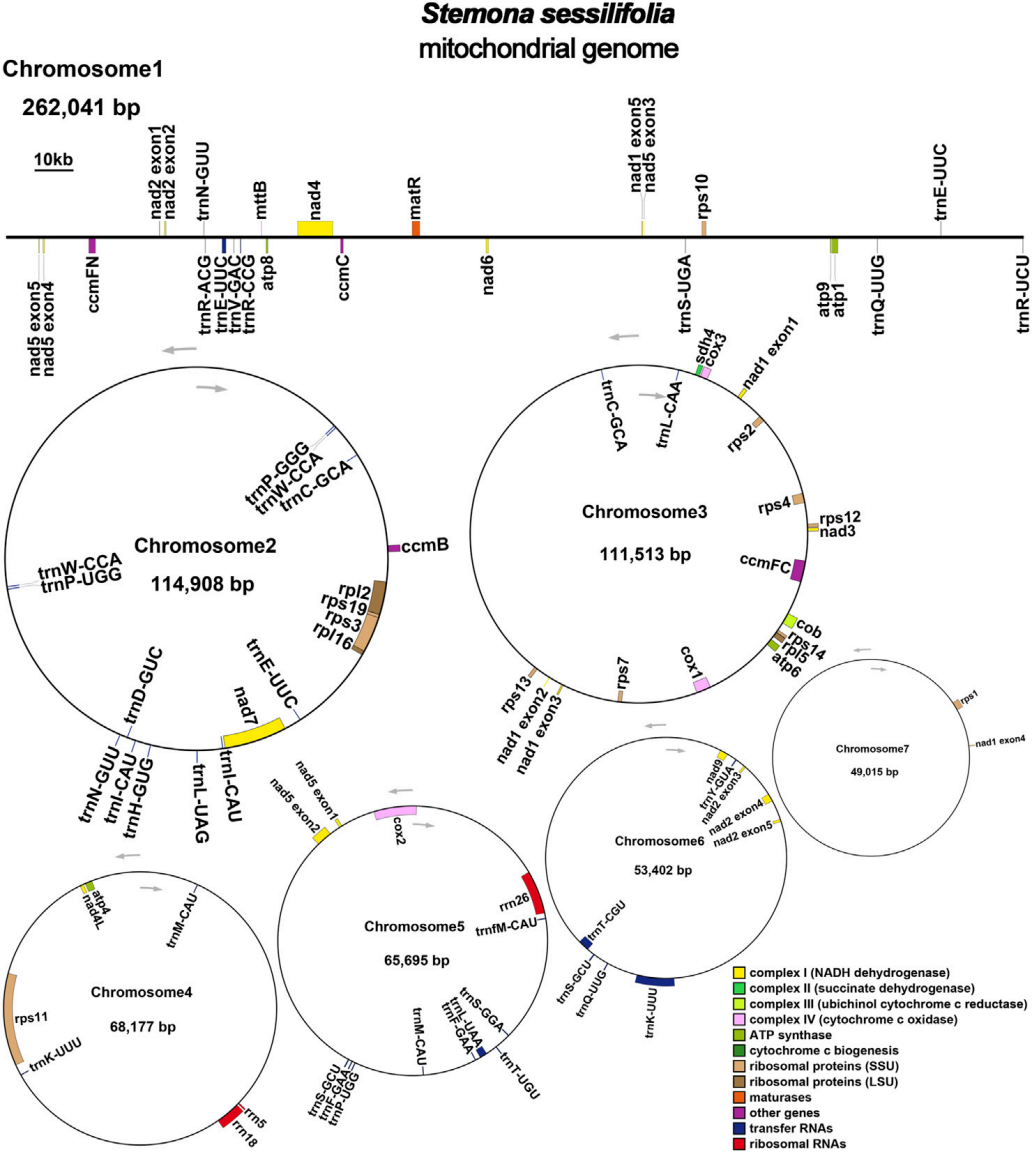
*S. sessilifolia* mitogenome gene map. Genes shown on the outside and inside of the circle are transcribed clockwise and counterclockwise, respectively.

**FIGURE 2:**
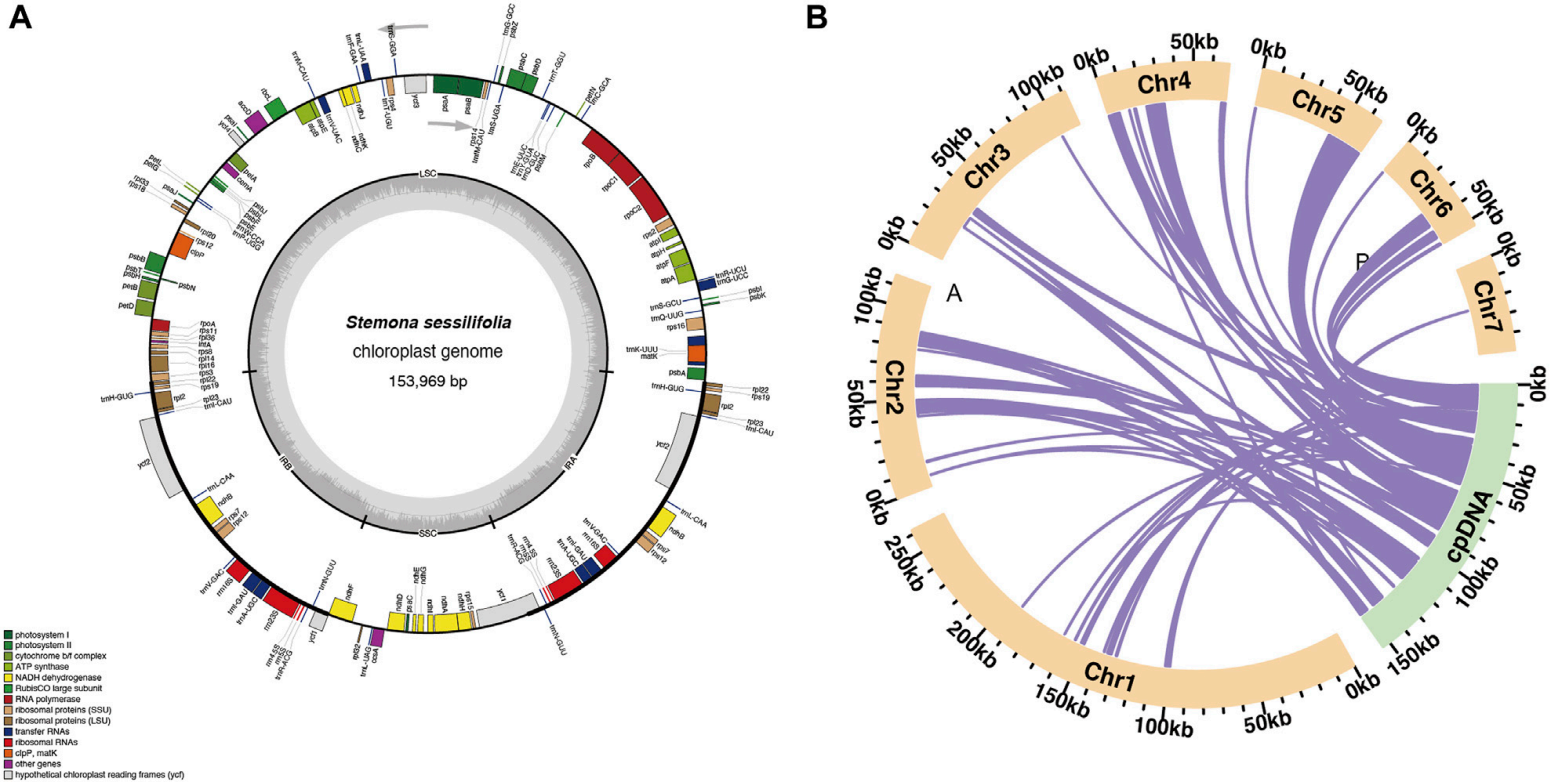
Schematic representation of homologous sequences between chloroplast genome and mitogenomes in *S. sessilifolia*. **(A)** *S. sessilifolia* chloroplast gene map. **(B)** The yellow arcs represent mitogenomes, the green arcs represent chloroplast genomes, and the purple lines between arcs correspond to homologous genome segments.

The mitochondrial genome of *S. sessilifolia* displays a branched structure (Supplementary Figure S1, Supplementary Data S1). After eliminating duplicated regions from the Nanopore data, we identified seven contigs, comprising one linear contig and six circular contigs. The total length of the mitogenome is 724,751 bp, with a GC content of 44.72% (Supplementary Table S1). We conducted annotation of the *S. sessilifolia* mitochondrial genome and identified a total of 39 protein-coding genes. Among these, 24 are unique mitochondrial core genes, while the remaining 15 are non-core genes. Additionally, there are 27 tRNA genes, with 11 of them being present in multiple copies, and 3 rRNA genes. The core genes consist of 5 ATP synthase genes (atp1, atp4, atp6, atp8, and atp9), 9 NADH dehydrogenase genes (nad1, nad2, nad3, nad4, nad4L, nad5, nad6, nad7, and nad9), 4 cytochrome C biogenesis genes (ccmB, ccmC, ccmFC, and ccmFN), 3 cytochrome C oxidase genes (cox1, cox2, and cox3), 1 protein transport subunit gene (mttB), 1 maturases gene (matR), and 1 ubiquinol-cytochrome C reductase gene (cob). Additionally, the non-core genes include 3 ribosomal large subunit genes (rpl2, rpl5, rpl16) and 11 ribosomal small subunit genes (rps1, rps2, rps3, rps4, rps7, rps10, rps11, rps12, rps13, rps14, and rps19). There is also 1 succinate dehydrogenase gene (sdh4) (Table 1); (Figure 1). Interestingly, several plastid genes were also annotated in the mtDNA, albeit mostly as fragments. These include rps7, rpoC2, rps15, accD, et al. This finding suggests a notable sequence migration was observed between the chloroplast DNA (cpDNA) and mtDNA of *S. sessilifolia*, which was accompanied by gene transfer, which will be discussed in detail below. Figure 2A presents the cpDNA genome map.

**TABLE 1:** Gene composition in the mitogenome of *S. sessilifolia*.

Group of genes	Name of genes
ATP synthase	*atp1, atp4 (×2), atp6, atp8, atp9*
NADH dehydrogenase	*nad1, nad2, nad3, nad4, nad4L, nad5, nad6 (×2), nad7, nad9 (×2)*
Cytochrome *b*	*cob (×2)*
Cytochrome *c* biogenesis	*cox1, cox2, cox3*
Maturases	*matR*
Protein transport subunit	*mttB*
Ribosomal protein large subunit	*rpl2, rpl5, rpl16*
Ribosomal protein small subunit	*rps1, rps2, rps3, rps4, rps7, rps10, rps11, rps12, rps13, rps14, rps19*
Succinate dehydrogenase	*sdh4*
Ribosome RNA	*rrn5, rrn18, rrn26*
Transfer RNA	*trnC-GCA(×2), trnD-GUC, trnE-UUC(×3), trnF-GAA(×2), trnfM-CAU, trnH-GUG, trnI-CAU (×2), trnK-UUU(×2), trnL-CAA, trnL-UAA, trnL-UAG, trnM-CAU(×2), trnN-GUU(×2), trnP-GGG, trnP-UGG(×2), trnQ-UUG (×2), trnR-ACG, trnR-CCG, trnR-UCU, trnS-GCU (×2), trnS-GGA, trnS-UGA, trnT-CGU, trnT- UGU, trnV-GAC, trnW-CCA (×2), trnY-GUA*

### 3.2 Chloroplast to mitochondrion DNA transformation

We observed significant sequence transfers from the chloroplast genome to the mitogenome in *S. sessilifolia*. Through sequence similarity analysis, we identified 42 homologous fragments between the mitogenome and chloroplast genome, totaling 95,262 base pairs. This fragment accounted for 13.14% of the entire mitogenome. Subsequently, we extracted and annotated these homologous sequences. Most of these fragments migrated from cpDNA to mtDNA, except for a few tRNA genes that exhibited high sequence similarity (Supplementary Table S6, Supplementary Data S2), making it challenging to determine the direction of migration (Figure 2B). Consequently, we denoted these sequences as mitochondrial plastid sequences (MTPTs), and detailed sequence information of MTPTs can be found in Supplementary Data S3. A total of 19 MTPTs with lengths exceeding 1,000 bp were identified: MTPT6, MTPT7, MTPT8, MTPT9, MTPT10, MTPT11, MTPT12, MTPT13, MTPT14, MTPT15, MTPT17, MTPT19, MTPT20, MTPT21, MTPT22, MTPT23, MTPT24, MTPT26, and MTPT27. The mapping results were visualized using the Tablet software (Milne et al., 2013), where a representative long read spanning the MTPT region was highlighted in deep blue (Supplementary Data S4), with the longest gene being MTPT27 spanning 20,203 bp. The annotation of these homologous sequences revealed the presence of 71 complete genes across the 42 homologous fragments. These included 52 protein-coding genes (PCGs) and 19 tRNA genes (trnC-GCA, trnF-GAA, trnG-UCC, trnH-GUG, trnI-CAU, trnK-UUU, trnL-CAA, trnL-UAA, trnL-UAG, trnM-CAU, trnM-CAU, trnN-GUU, trnP-UGG, trnQ-UUG, trnS-GCU, trnS-GGA, trnT-UGU, trnV-UAC, trnW-CCA). For detailed information on the transferred genes, please refer to Supplementary Table S2.

### 3.3 The prediction of RNA editing

**FIGURE 3:**
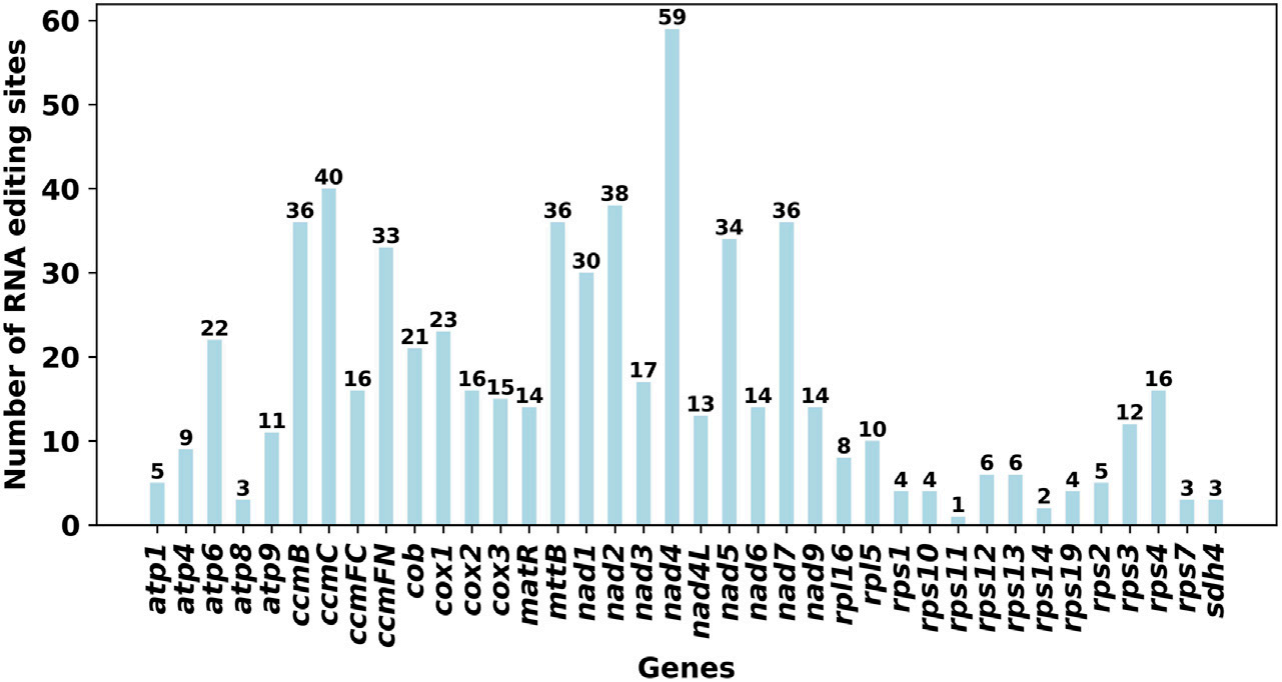
Characteristics of the RNA editing sites identified in PCGs of *S. sessilifolia* mitogenome. Number of RNA editing sites predicted by individual PCGs using Deepred-mt. The abscissa shows the name of the gene, and the ordinate shows the number of edited sites.

Using Deepred-mt, we identified a total of 639 potential RNA editing sites on 32 unique protein-coding genes (PCGs) in the mitogenome, with cutoff value of 0.9 as the criterion. Interestingly, all identified edits were C-U conversions. Among these mitochondrial genes, the gene nad4 displays the highest number of editing sites with 59 occurrences, followed closely by the ccmC gene with 40 editing sites (Figure 3).

### 3.4 Synteny analysis and phylogenetic inference

**FIGURE 4:**
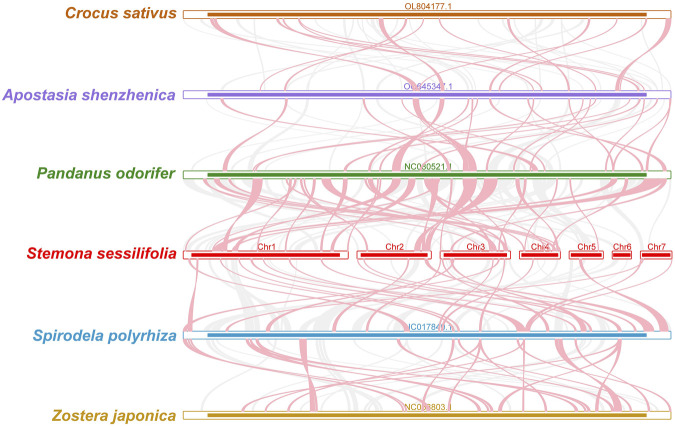
Mitogenome synteny. Bars indicated the mitogenomes, and the ribbons showed the homologous sequences between the adjacent species. The red areas indicate where the inversion occurred, the gray areas indicate regions of good homology. Common blocks less than 0.5 kb in length are not retained, and regions that fail to have a common block indicate that they are unique to the species.

To investigate the synteny relationship between *S. sessilifolia* and closely related species, we utilized MCscanXto generate multiple synteny plots based on the sequence similarity. Figure 4 illustrates that the co-linear blocks exhibit varying arrangements across individual mitochondrial genomes. Although a substantial number of blocks were detected between *S. sessilifolia* and *Pandanus odorifer* and *Spirodela polyrhiza*, these co-linear blocks appeared to be shorter in length. Additionally, unique sequences specific to *S. sessilifolia* were also identified, which was lacking homology with other species. These findings indicate extensive genomic rearrangements within the *S. sessilifolia* mitogenome, leading to an exceptionally unconserved mitochondrial structure.

**FIGURE 5:**
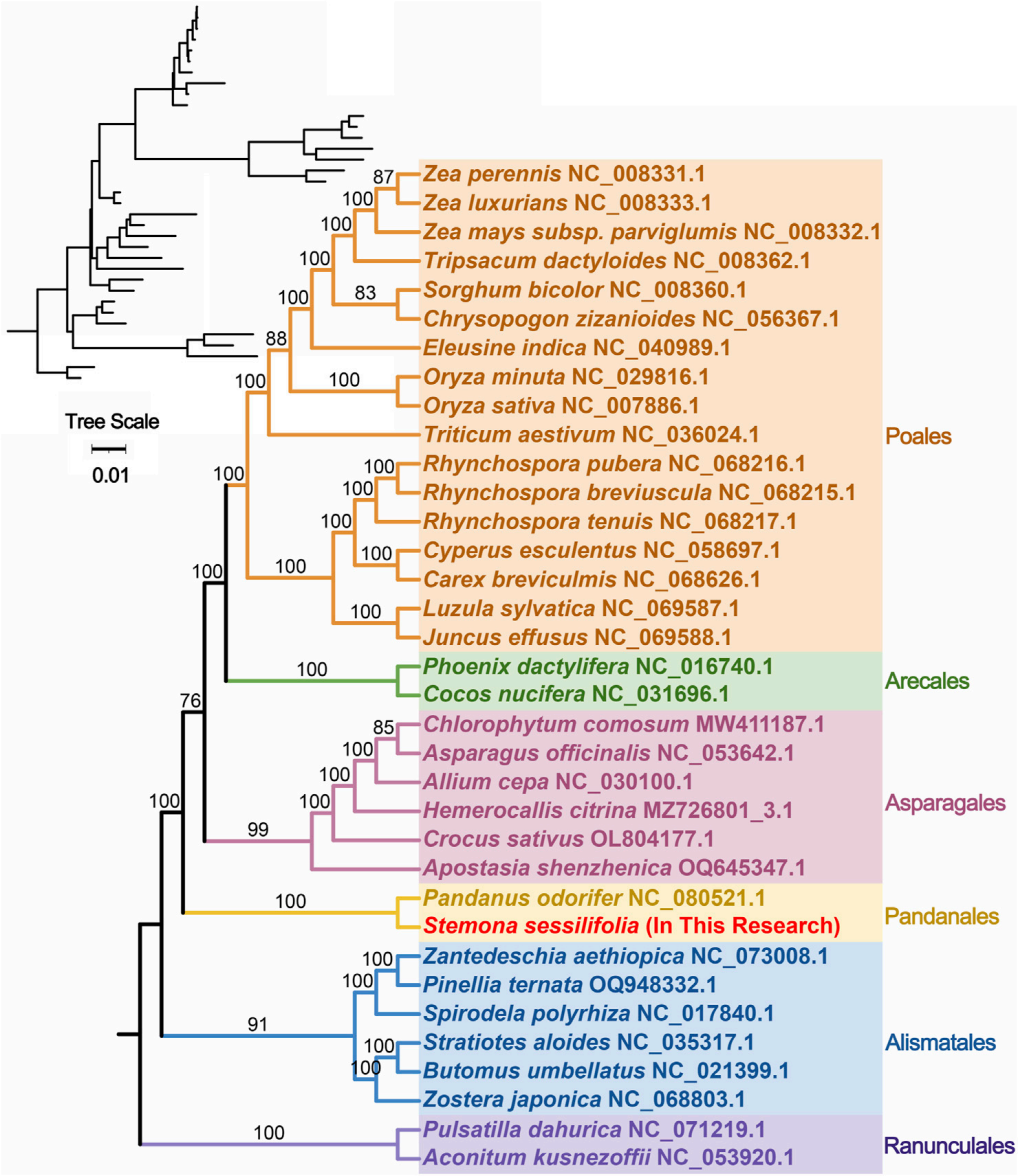
Phylogenetic tree of 35 angiosperms based on the sequences of 24 conserved mitochondrial PCGs. Two Ranunculales species were chosen as the outgroup. The number at each node is the bootstrap probability.

To further explore the evolutionary relationships of *S. sessilifolia* mitochondria, we constructed a phylogenetic tree using the DNA sequences of 24 conserved mitochondrial PCGs (atp1, atp4, atp6, atp8, atp9, ccmB, ccmC, ccmFC, ccmFN, cob, cox1, cox2, cox3, matR, mttB, nad1, nad2, nad3, nad, nad4L, nad5, nad6, nad7, nad9) from 35 species across 6 orders of angiosperms. Two mitochondrial genomes from Ranunculales species were designated as outgroups (Supplementary Table S3). The topology of the phylogenetic tree based on mitochondrial DNA aligned with the current classification of the Angiosperm Phylogeny Group (APG). *S. sessilifolia* was classified within the family Stemonaceae, under the order Pandanales, illustrating its close evolutionary relationship with *P. odorifer* (Figure 5).

### 3.5 PCGs codon usage analysis

**FIGURE 6:**
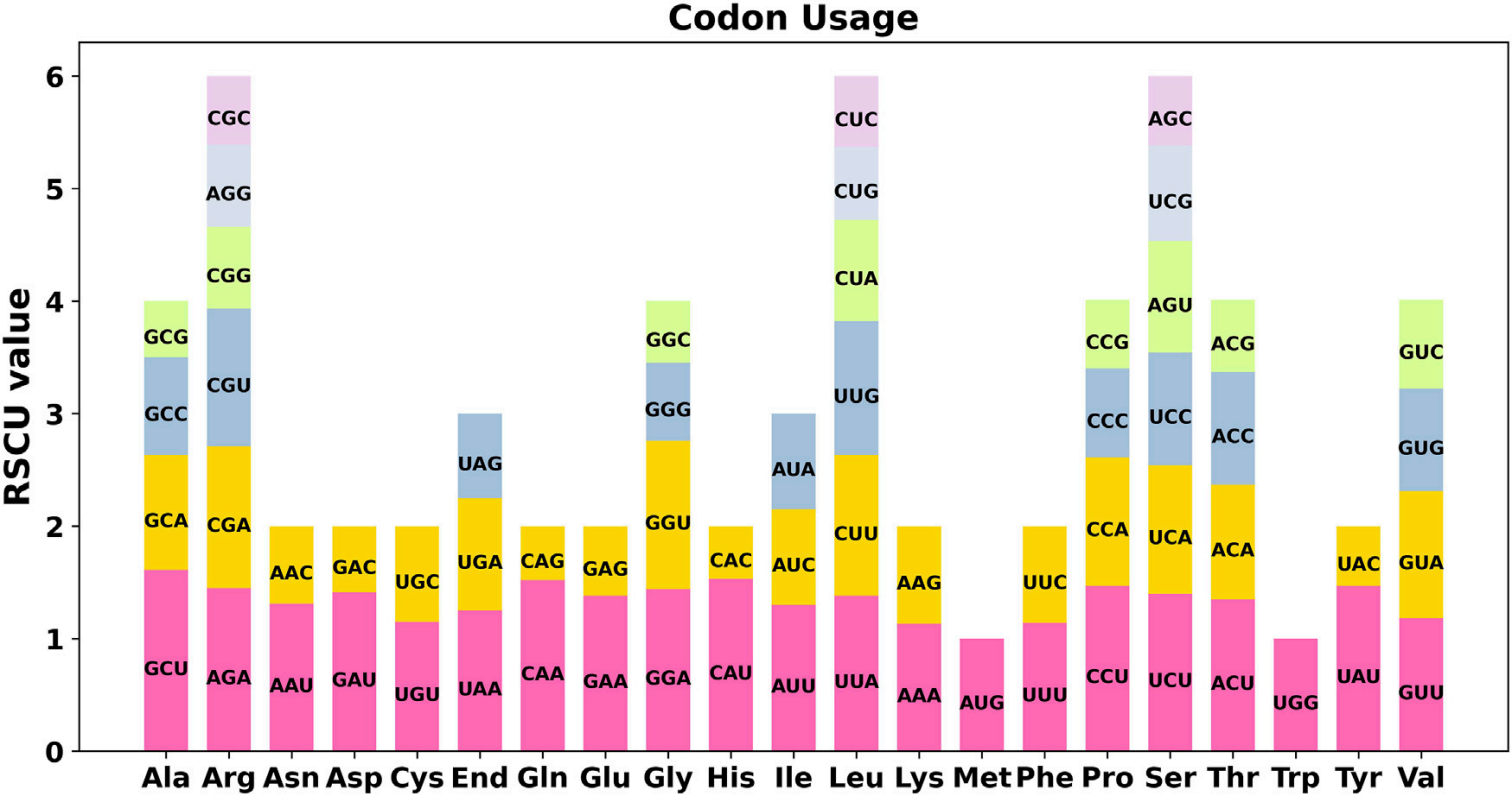
*S. sessilifolia* mitogenome relative synonymous codon usage (RSCU). Codon families are shown on the *x*-axis. RSCU values are the number of times a particular codon is observed relative to the number of times that codon would be expected for a uniform synonymous codon usage.

We conducted a codon usage analysis of the 39 unique protein-coding genes (PCGs) in *S. sessilifolia.* The codon usage for each amino acid is presented in Supplementary Table S4. Relative synonymous codon usage (RSCU) values greater than 1 signify a preference for specific amino acids. Among the mitochondrial PCGs, apart from the start codon AUG (Met) and UGG (Trp), there was a noticeable preference for certain codons (Figure 6). For example, alanine (Ala) showed a higher preference for the codon GCU, evidenced by an RSCU value of 1.61, which was the highest observed. Similarly, histidine (His) exhibited a preference for the codon CAU, with an RSCU value of 1.53.

### 3.6 *S. sessilifolia* mitogenome repeats analysis

**FIGURE 7:**
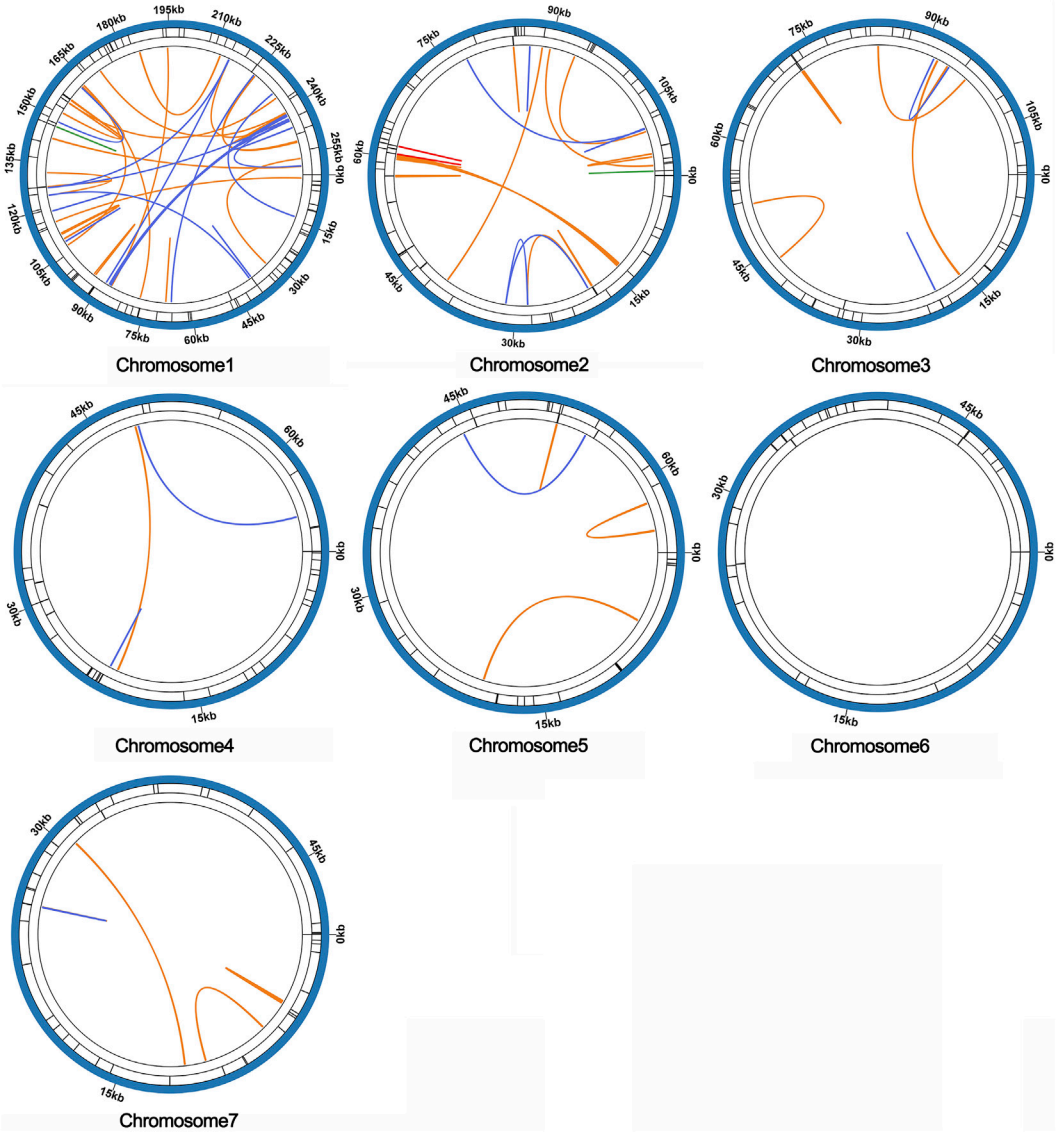
The distribution of repeats in the mitogenome of *S. sessilifolia*. The inner circle shows the dispersed repeats with a length greater than or equal to 50 bp, in which blue represents palin-dromic repeats, orange represents forward repeats, red represents reverse repeats and green rep-resents complementary repeats. The black line on the second circle represents tandem repeats, and the black line on the outermost circle represents microsatellite repeats, respectively.

Microsatellites, also known as simple repeat sequences (SSRs), were analyzed to determine the presence of repeat sequences in the mitogenome. The results indicated that chromosomes 1-7 of *S. sessilifolia* contained 85, 56, 51, 34, 36, 33, and 40 SSRs, respectively. Monomeric polymers constituted the largest proportion in almost all chromosomes (Supplementary Table S5). Additionally, there were 15 and 13 tandem repeats in chromosomes 1 and 2, respectively, while other chromosomes had fewer than 5 tandem repeats. Dispersed repeats with a length greater than or equal to 30 bp were detected in each chromosome except for chromosome 6. Among these dispersed repeats, chromosomes 1 through 5 and 7 contained palindromic repeats in the following numbers: 35, 5, 2, 2, 1, and 1, respectively. Additionally, they contained forward repeats in the quantities of 36, 23, 10, 1, 4, and 5, respectively. Chromosomes 1 and 2 carried 3 reverse repeats, 3 complementary repeats, along with 1 additional complementary repeat. Overall, the mitogenome of *S. sessilifolia* included 335 SSRs, 135 pairs of dispersed repeats, and 43 tandem repeats. A circular diagram in Figure 7 illustrates the different types of repeat sequences.

## 4 Discussion

In this study, we conducted an extensive characterization of the mitochondrial genome of *S. sessilifolia* and investigated a range of genetic features. Notably, we uncovered a phenomenon known as chloroplast-to-mitochondrion DNA transformation, wherein DNA sequences are transferred from the chloroplast genome to the mitogenome. This phenomenon has also been observed in other Chinese herbal medicines such as *C. chinensis, C. deltoidei, C. omeiensis*, and *Saposhnikovia* divaricate (Ni et al., 2022; Zhong et al., 2023). By examining mitogenome, we identified 42 homologous fragments between the mitogenome and chloroplast genome, accounting for 13.14% of the total mitogenome. These fragments, termed mitochondrial plastid sequences (MTPTs), encompass complete genes within them. The presence of similar transferred genes has been documented in numerous mitochondrial genomes. For instance, Quercus acutissima contains 15.7 kb MTPTs, representing 3.49% of its mitogenome (Liu et al., 2022), while *S. miltiorrhiza* exhibits sixteen fragments resembling the plastome in its mitogenome (Yang et al., 2022). Such transferred genes highlight the dynamic nature of plant mitochondrial genomes and suggest the inter-organelle genetic exchange.

Using Deepred-mt, we predicted RNA editing sites in the *S. sessilifolia* mitogenome. A total of 639 C-to-U RNA editing sites were identified across 32 unique mitochondrial PCGs. Among all genes, nad4 and ccmC had the highest number of editing sites. The Nad4 gene encodes mitochondrial respiratory chain complex I subunit IV and is highly conserved in maize mitochondria (Marienfeld and Newton, 1994). Prior studies have established a connection between DEK43 and cis-splicing of Nad4 gene in maize mitochondria (Ren et al., 2020), and the significance of Nad4 intron 3 in normal seed development has also been demonstrated (Zhu et al., 2019). CcmC plays a pivotal role in the maturation of cytochrome c and is translated as a long precursor with an N-extension. Our analysis identified 31 C-to-U RNA editing events in N-extension and cmC-homologous region (ccmC-core region), which contribute to the conservation of amino acid sequence (Kitazaki et al., 2009). RNA editing serves as a crucial post-transcriptional modification mechanism which greatly influence the expression and function of mitochondrial gene.

Through synteny analysis and phylogenetic inference, we explored the evolutionary of *S. sessilifolia* mitochondria. The co-linear blocks observed in the mitogenomes of closely related species did not exhibit the same arrangement, indicating extensive genomic rearrangements. Our phylogenetic analysis unveiled that *S. sessilifolia* belongs to the family Stemonaceae within the order Pandanales, showing a close relationship to *P. odorifer*.

An analysis of codon usage among the mitochondrial PCGs in *S. sessilifolia* demonstrated preferential codon usage for certain amino acids. For instance, alanine (Ala) displayed a higher preference for the codon GCU, whereas histidine (His) favored the codon CAU. Comprehending these codon usage patterns enhances our understanding of the molecular evolution and functional constraints associated with mitochondrial genes.

Repeat sequences played a significant role in the rearrangement of the mitogenome (Cole et al., 2018). Extensive gene repeat sequences have been observed in Stemonaceae species, including *S. mairei* (Lu et al., 2018a), *C. japonica* (Lu et al., 2018b), and *S. parviflora* (Wei and Li, 2022). The repeats analysis indicated the presence of microsatellites (SSRs), tandem repeats, and dispersed repeats within the *S. sessilifolia* mitogenome. SSRs were the most abundant repeat type, with monomeric polymers being the most prevalent. Dispersed repeats and tandem repeats exhibited variations in their distribution across different chromosomes.

In conclusion, our study provides a thorough analysis of the mitochondrial genome of *S. sessilifolia*. The identification of transferred genes from the chloroplast genome, RNA editing sites, evolutionary relationships, codon usage patterns, and repeat sequences enhances our understanding of the genetic features and evolutionary dynamics of *S. sessilifolia*. Future investigations could focus on functional analyses of the transferred genes and on unraveling the impact of RNA editing on mitochondrial gene expression in *S. sessilifolia*.

## Data Availability

The datasets presented in this study can be found in online repositories. The names of the repository/repositories and accession number(s) can be found below: https://www.ncbi.nlm.nih.gov/, PP234475.1, https://www.ncbi.nlm.nih.gov/, PRJNA1073764, https://www.ncbi.nlm.nih.gov/, SAMN39839158, https://www.ncbi.nlm.nih.gov/, SRR27999910, https://www.ncbi.nlm.nih.gov/genbank/, PP692484, https://www.ncbi.nlm.nih.gov/genbank/, PP692485, https://www.ncbi.nlm.nih.gov/genbank/, PP692486, https://www.ncbi.nlm.nih.gov/genbank/, PP692487, https://www.ncbi.nlm.nih.gov/genbank/, PP692488, https://www.ncbi.nlm.nih.gov/genbank/, PP692489, https://www.ncbi.nlm.nih.gov/genbank/, PP692490.
