# Supplementary figures and images for: Mitochondrial genome complexity in Stemona sessilifolia: nanopore sequencing reveals chloroplast gene transfer and DNA rearrangements
Source: Front Genet. 2024 Jun 4;15:1395805. doi: 10.3389/fgene.2024.1395805 (PMC11188483; doi:10.3389/fgene.2024.1395805)

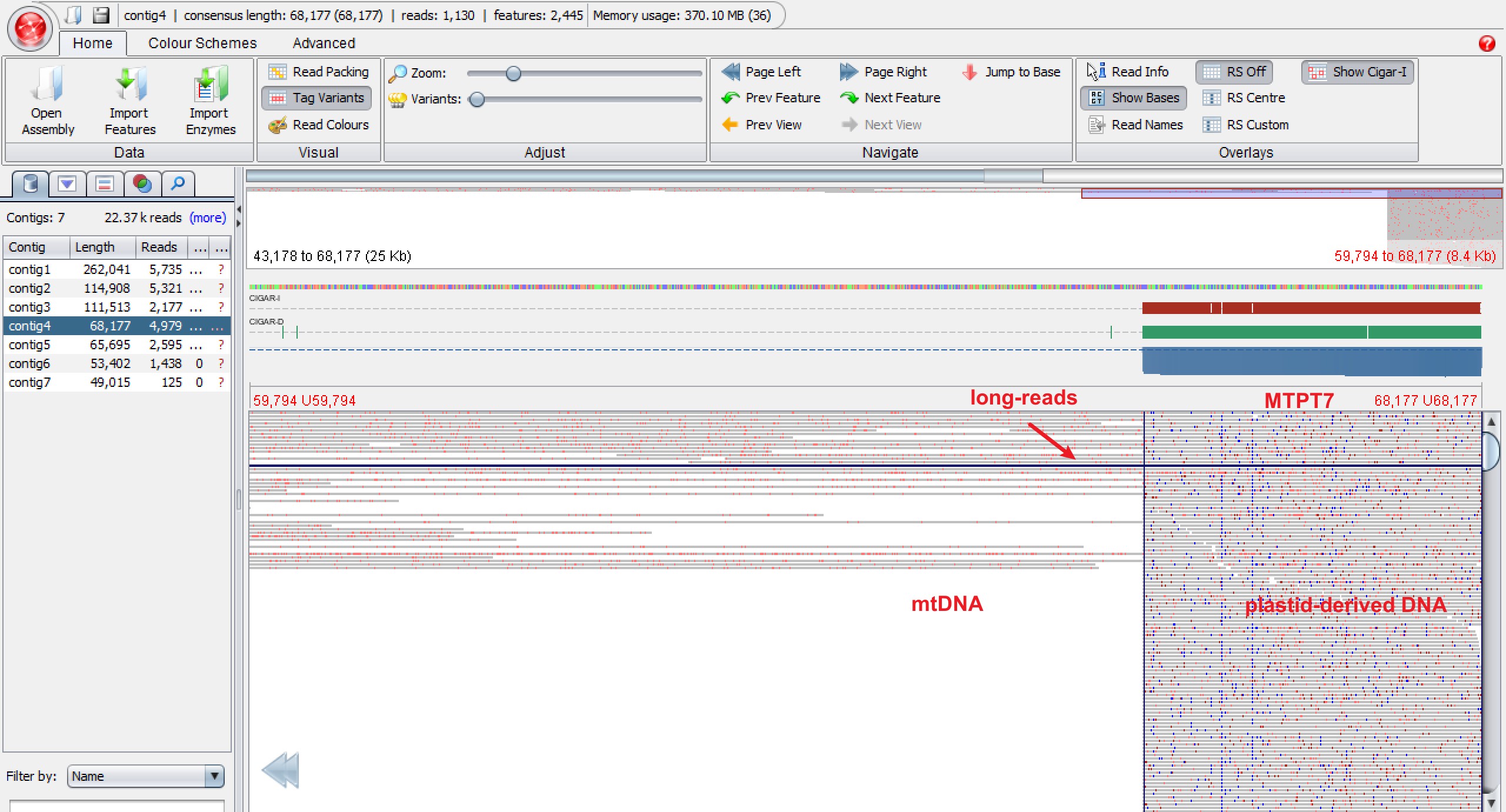

Supplement: Supplementary file 1 [file DataSheet1.ZIP › Supplementary Data/Supplementary Data4/MTPT7.jpg]

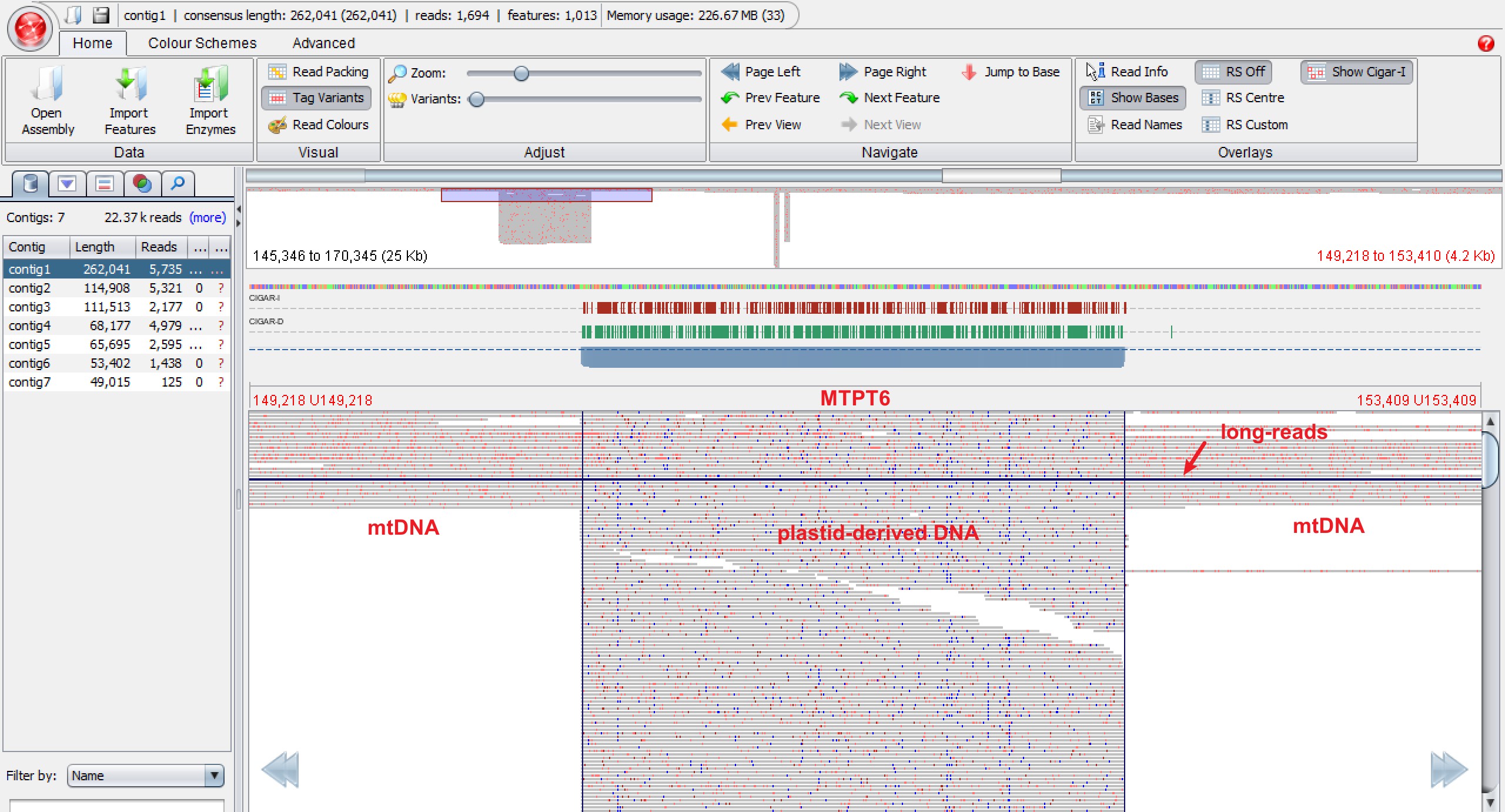

Supplement: Supplementary file 1 [file DataSheet1.ZIP › Supplementary Data/Supplementary Data4/MTPT6.jpg]

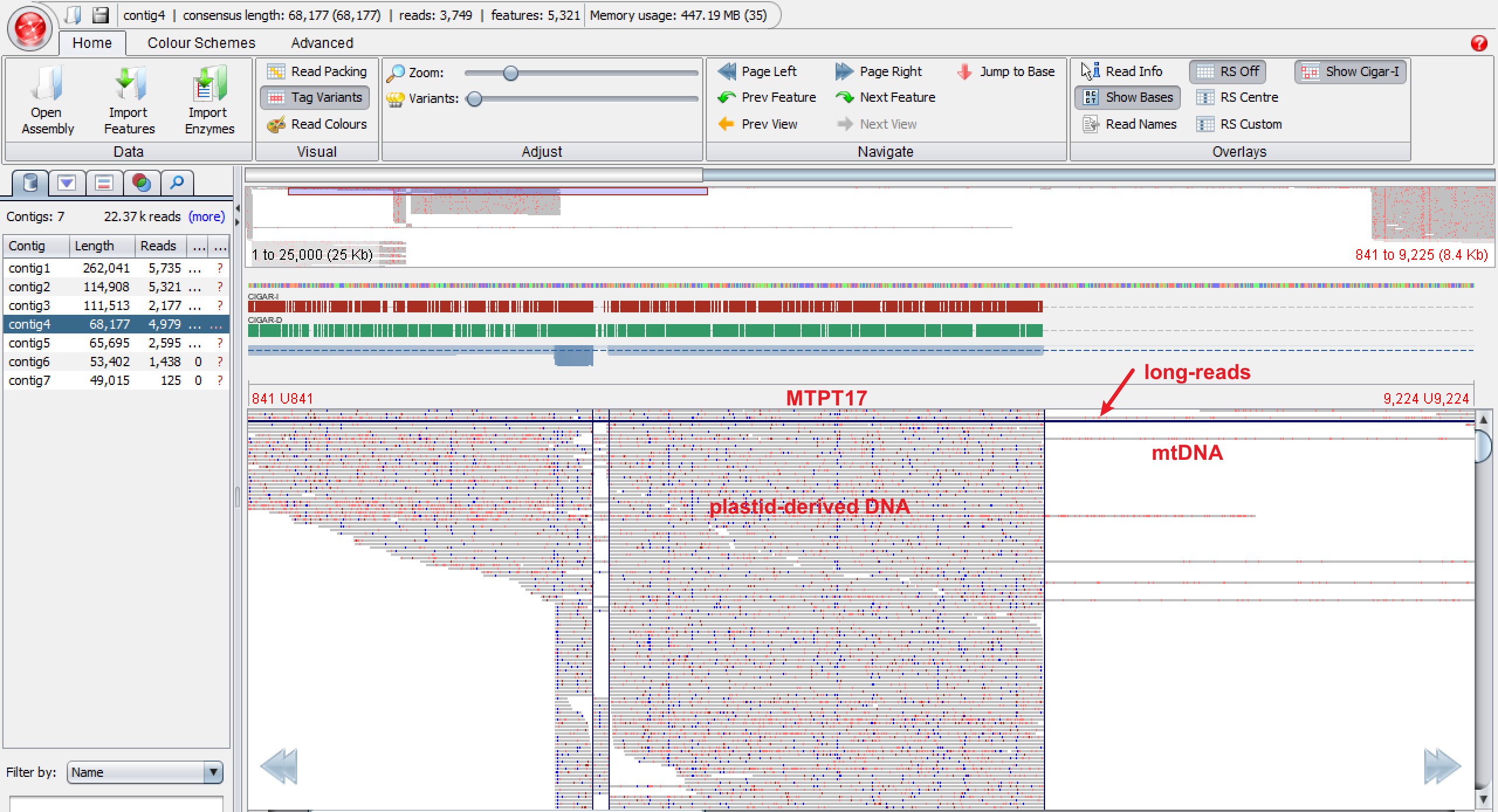

Supplement: Supplementary file 1 [file DataSheet1.ZIP › Supplementary Data/Supplementary Data4/MTPT17.jpg]

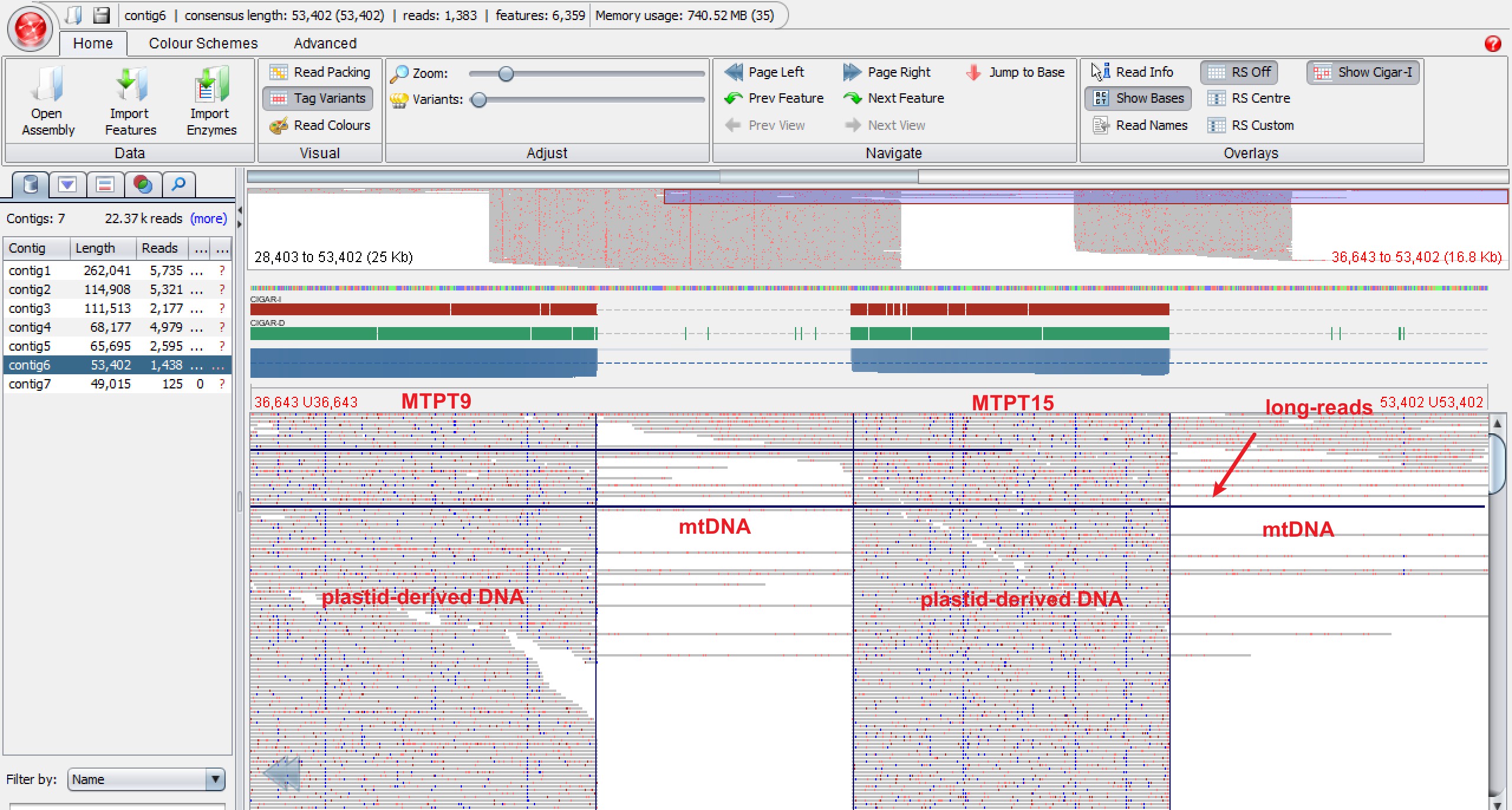

Supplement: Supplementary file 1 [file DataSheet1.ZIP › Supplementary Data/Supplementary Data4/MTPT15.jpg]

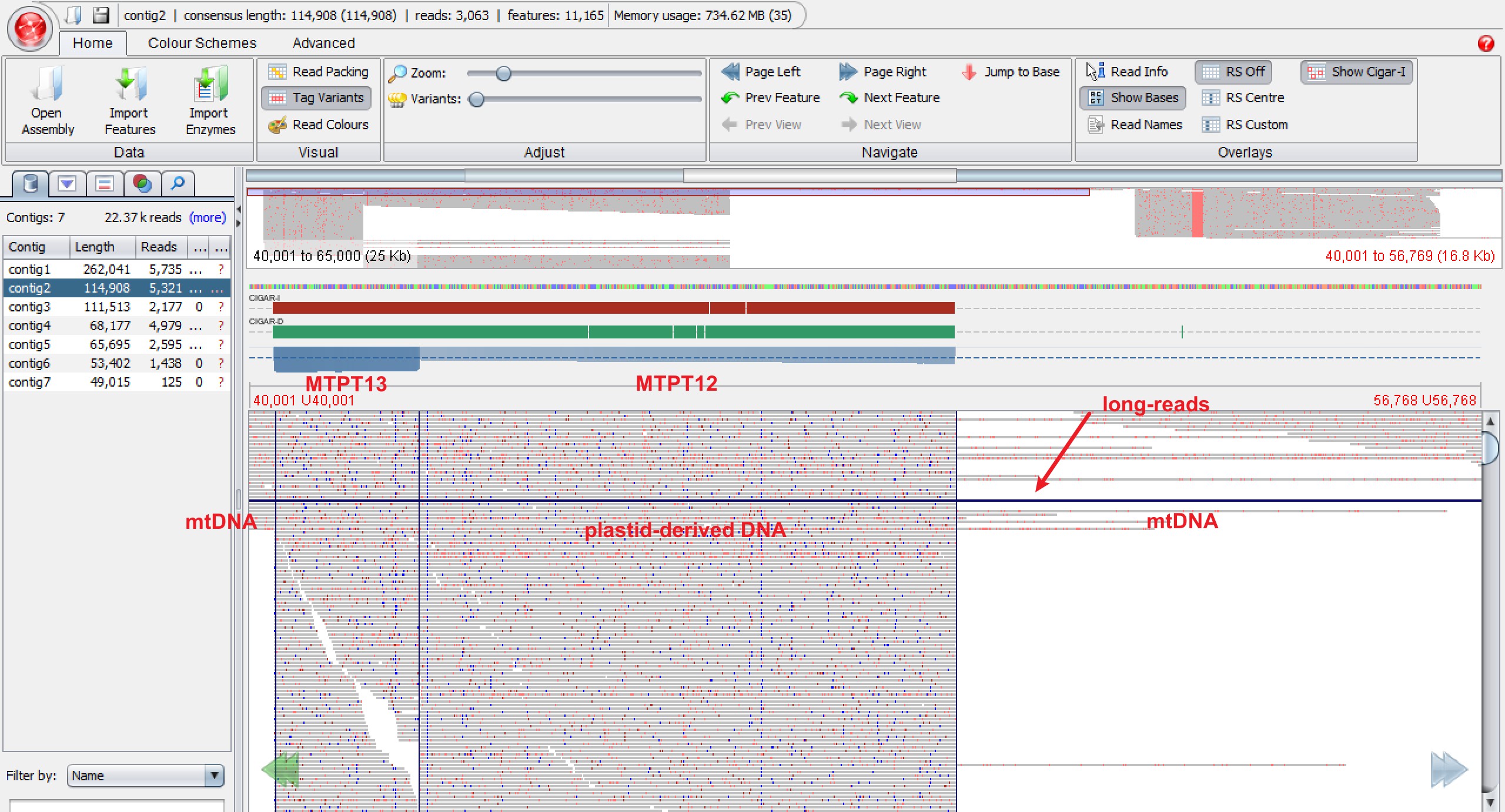

Supplement: Supplementary file 1 [file DataSheet1.ZIP › Supplementary Data/Supplementary Data4/MTPT12-MTPT13.jpg]

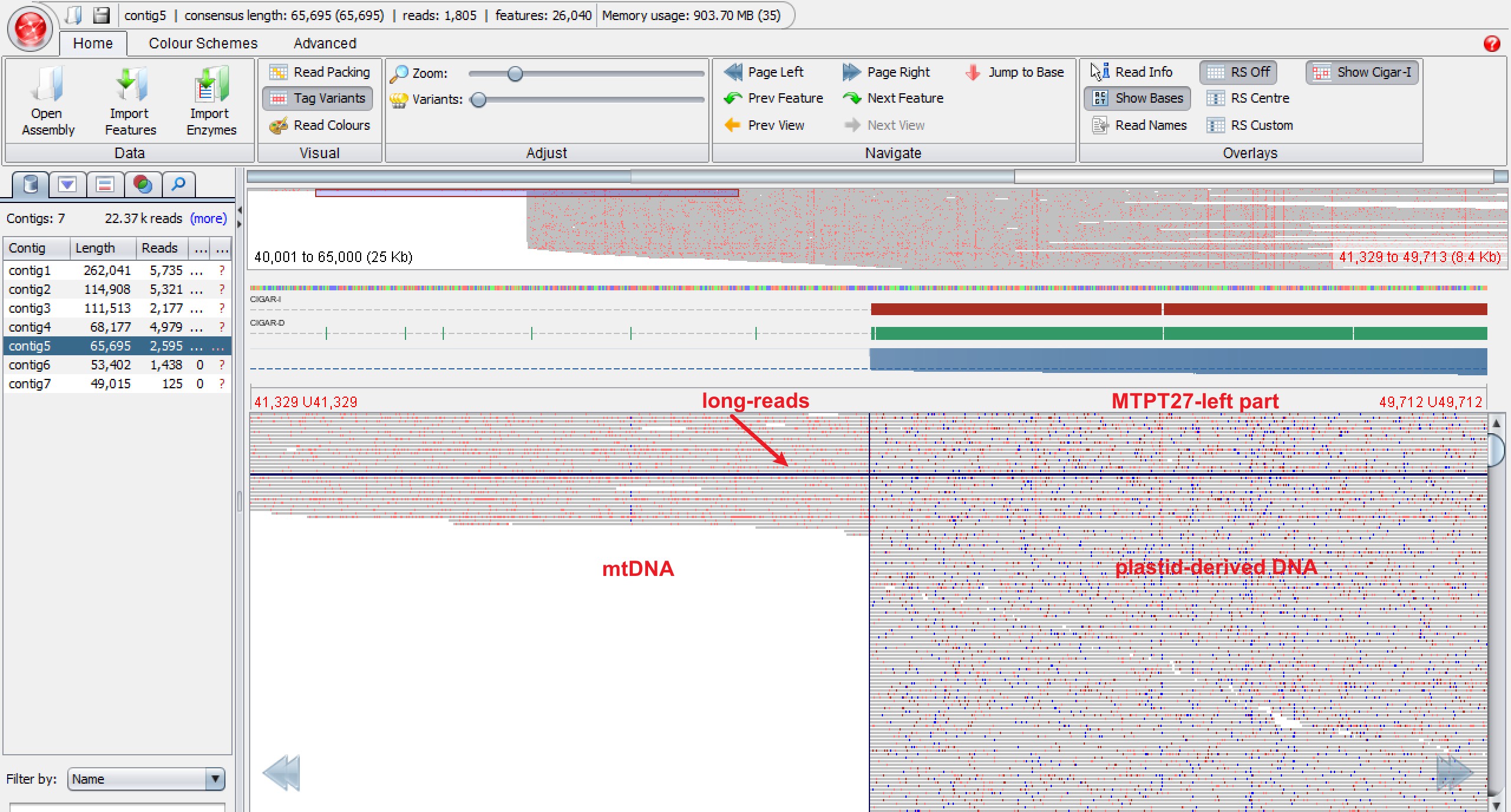

Supplement: Supplementary file 1 [file DataSheet1.ZIP › Supplementary Data/Supplementary Data4/MTPT27-left.jpg]

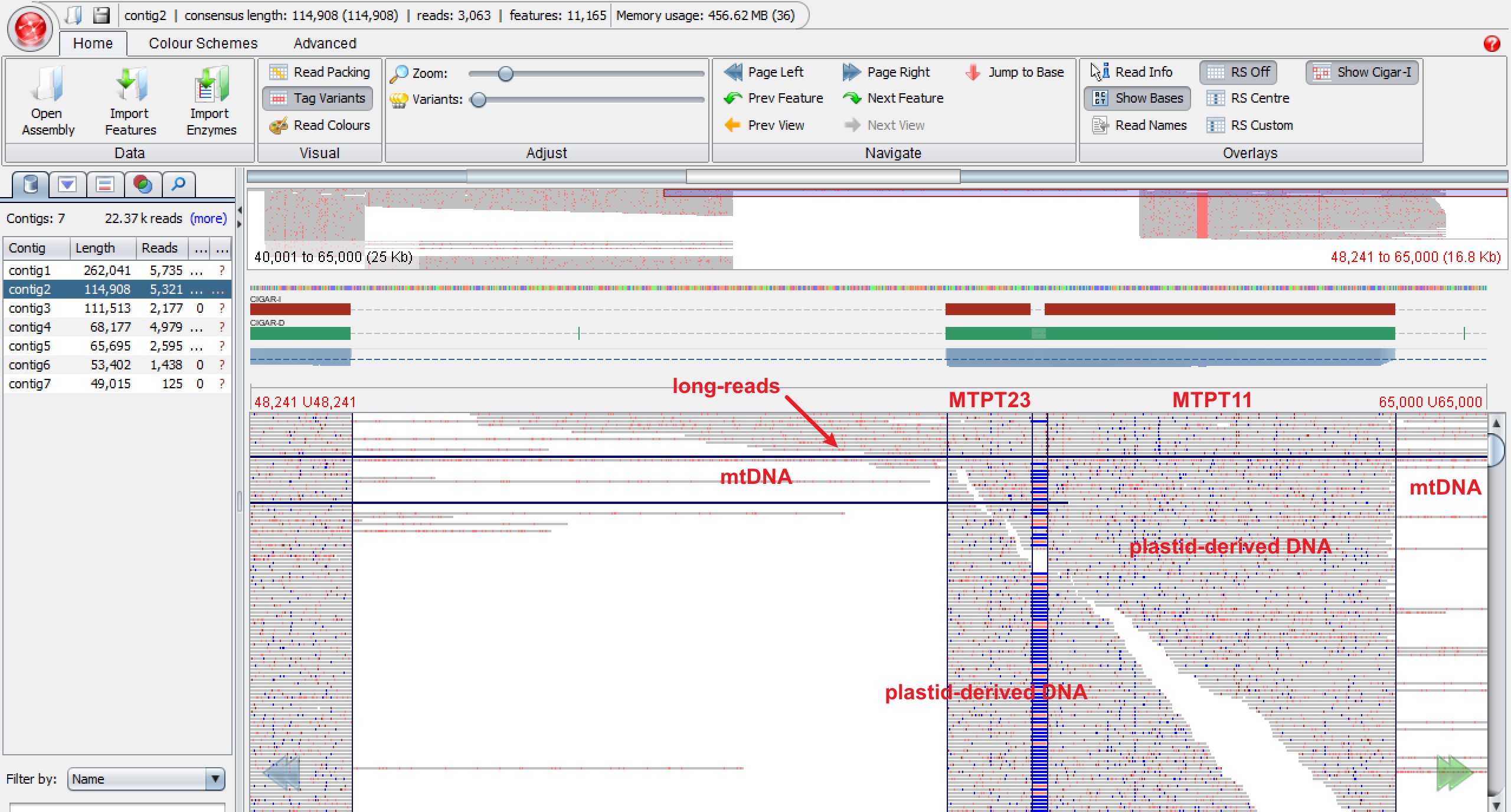

Supplement: Supplementary file 1 [file DataSheet1.ZIP › Supplementary Data/Supplementary Data4/MTPT11-MTPT23.jpg]

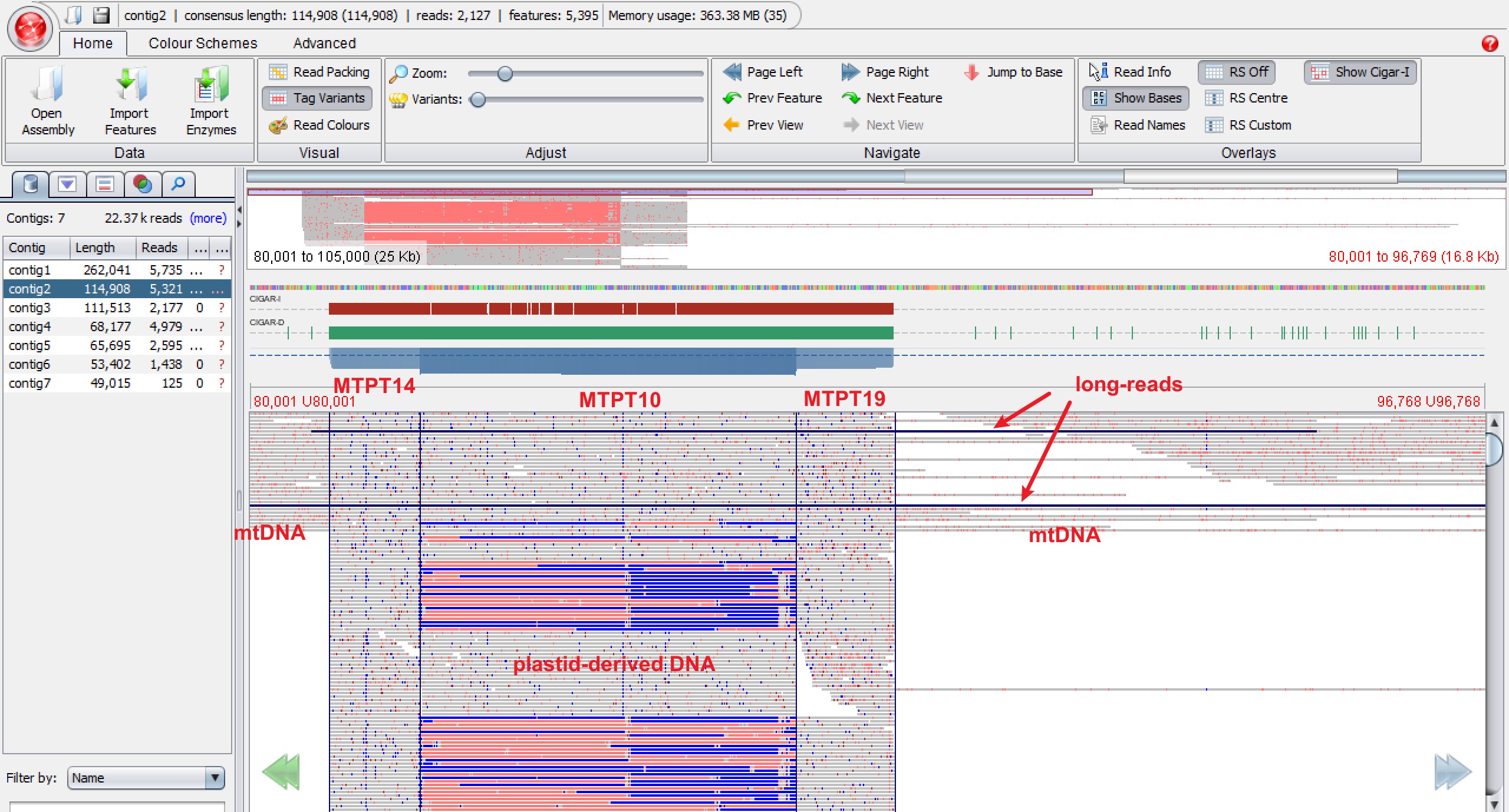

Supplement: Supplementary file 1 [file DataSheet1.ZIP › Supplementary Data/Supplementary Data4/MTPT10-MTPT14-MTPT19.jpg]

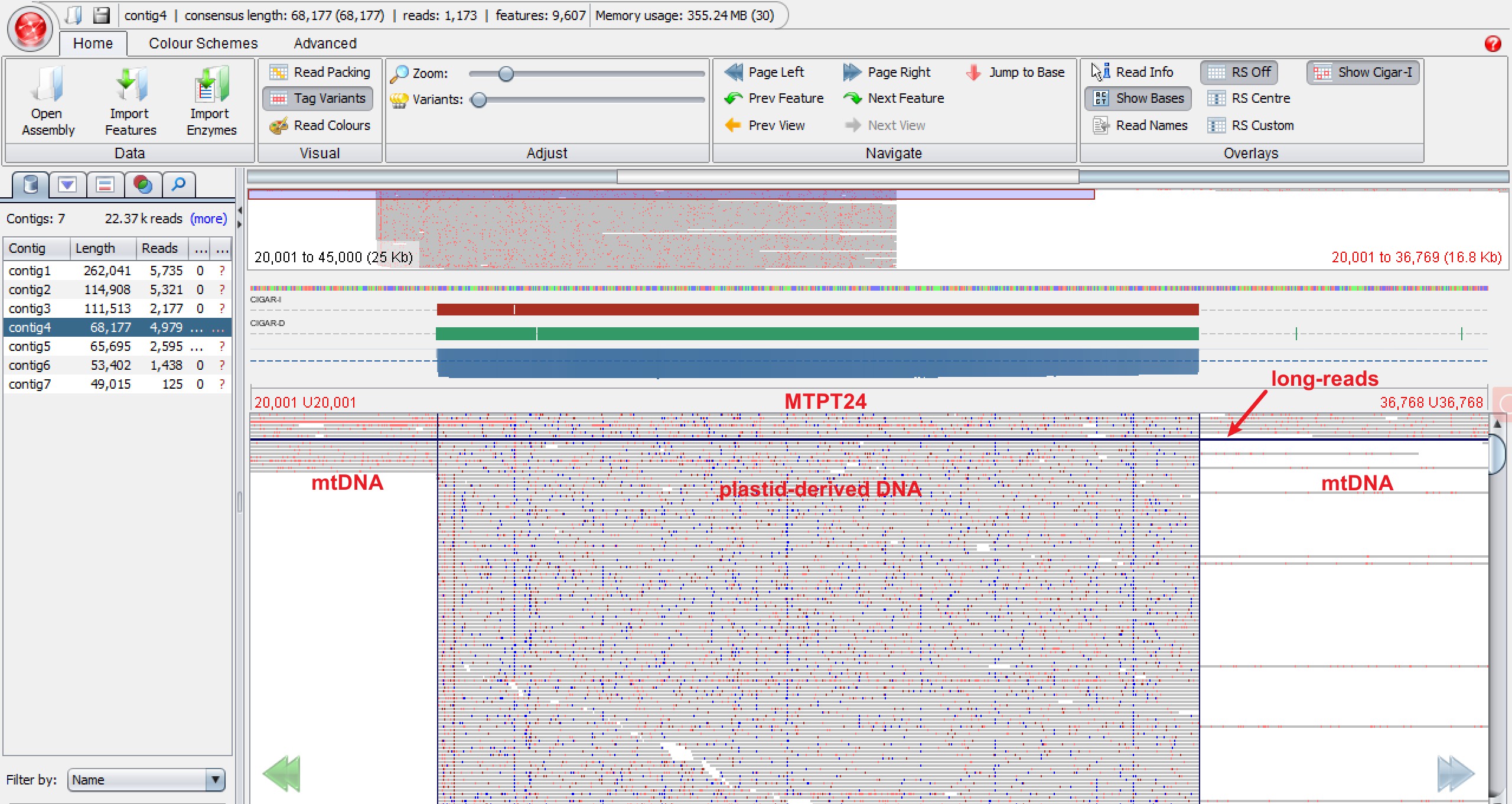

Supplement: Supplementary file 1 [file DataSheet1.ZIP › Supplementary Data/Supplementary Data4/MTPT24.jpg]

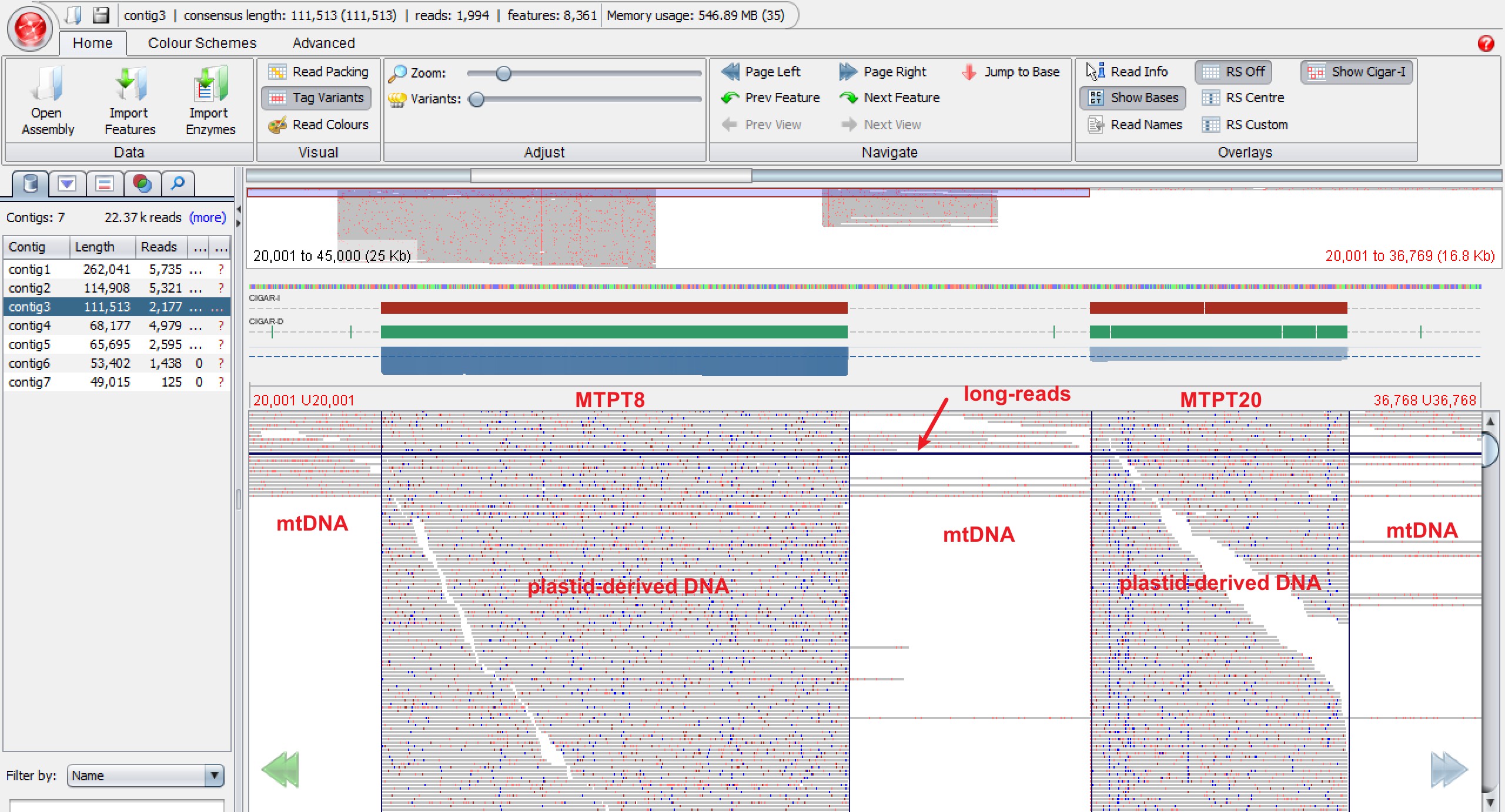

Supplement: Supplementary file 1 [file DataSheet1.ZIP › Supplementary Data/Supplementary Data4/MTPT8-MTPT20.jpg]

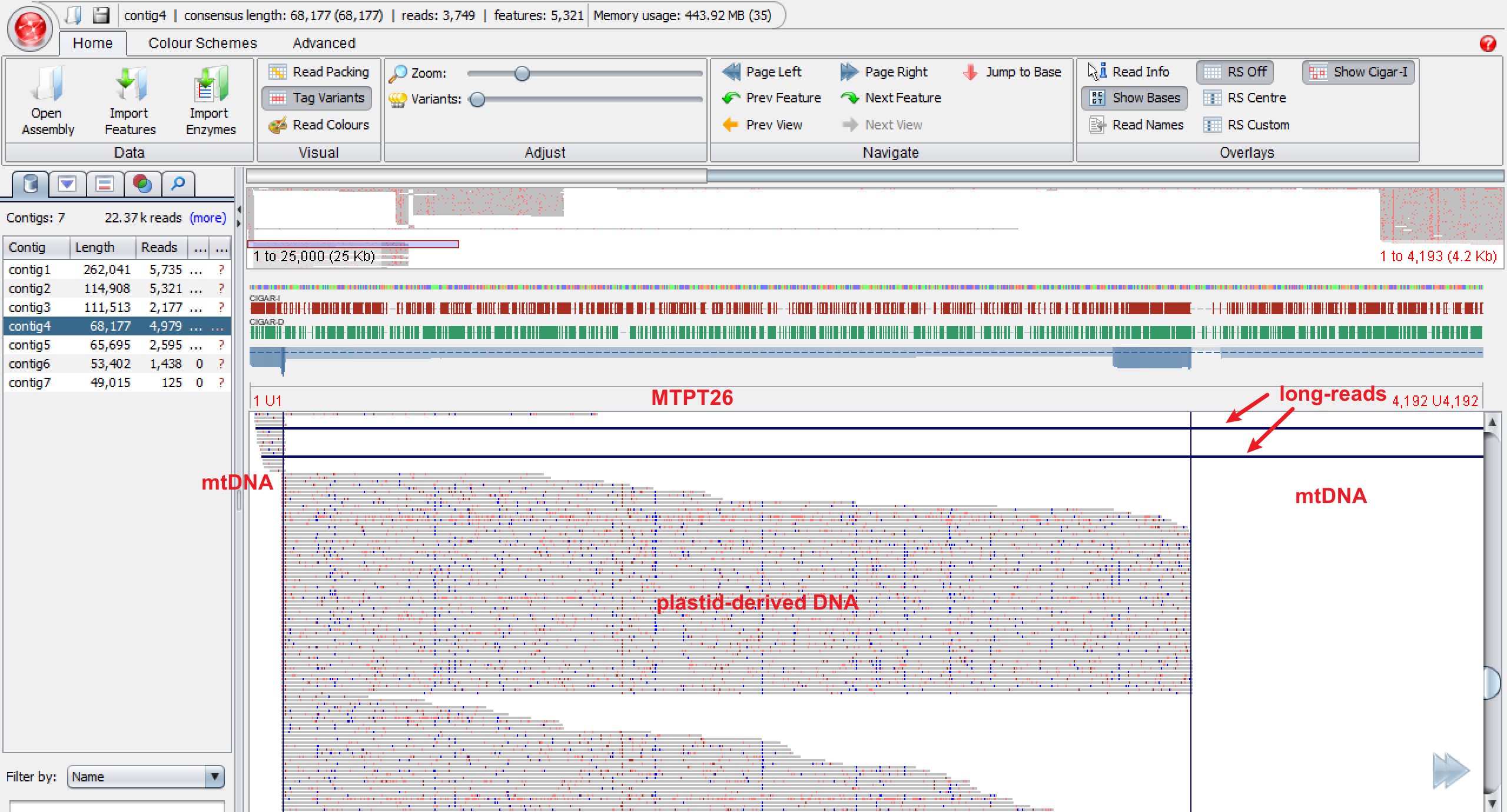

Supplement: Supplementary file 1 [file DataSheet1.ZIP › Supplementary Data/Supplementary Data4/MTPT26.jpg]

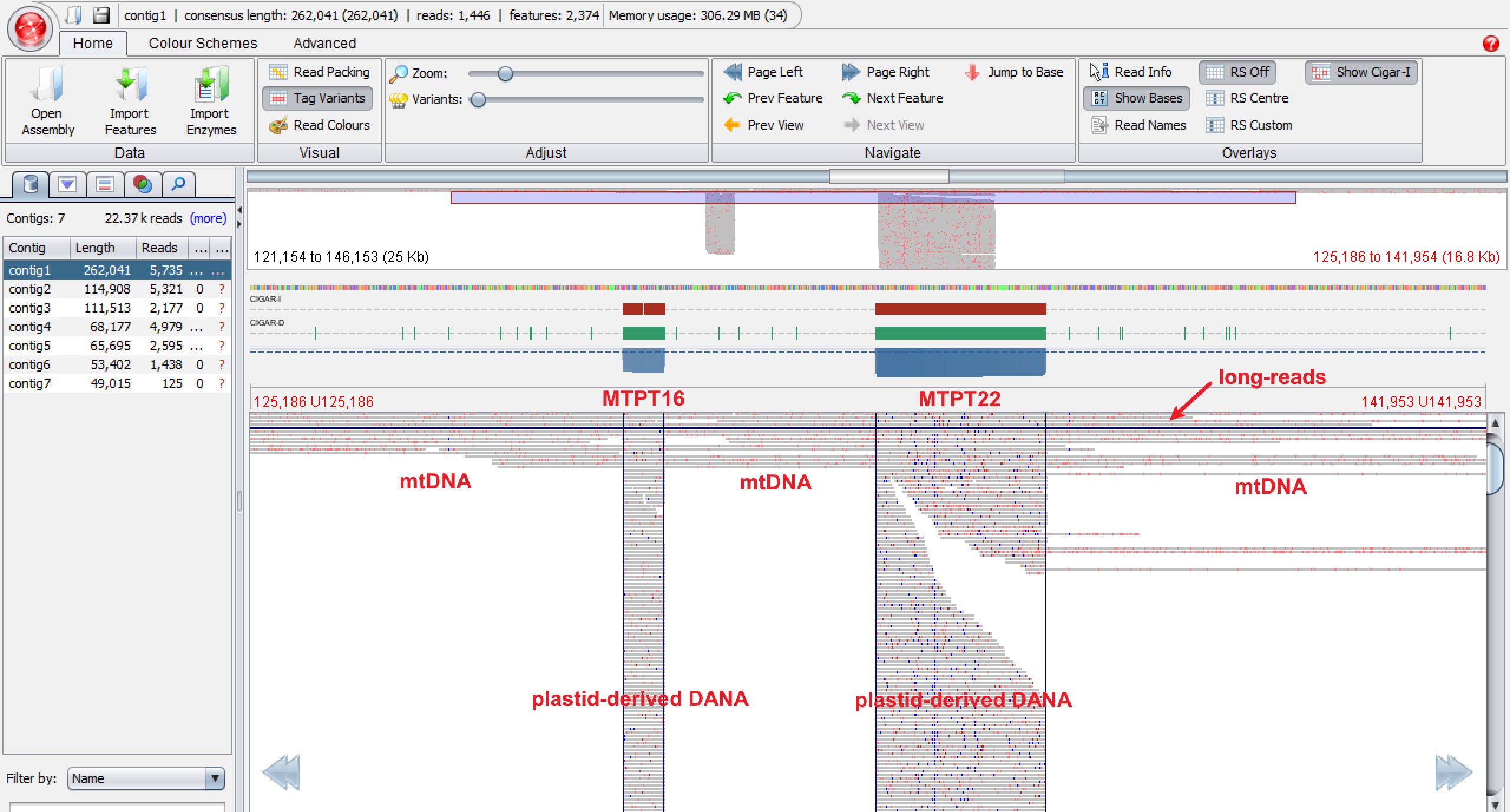

Supplement: Supplementary file 1 [file DataSheet1.ZIP › Supplementary Data/Supplementary Data4/MTPT22.jpg]

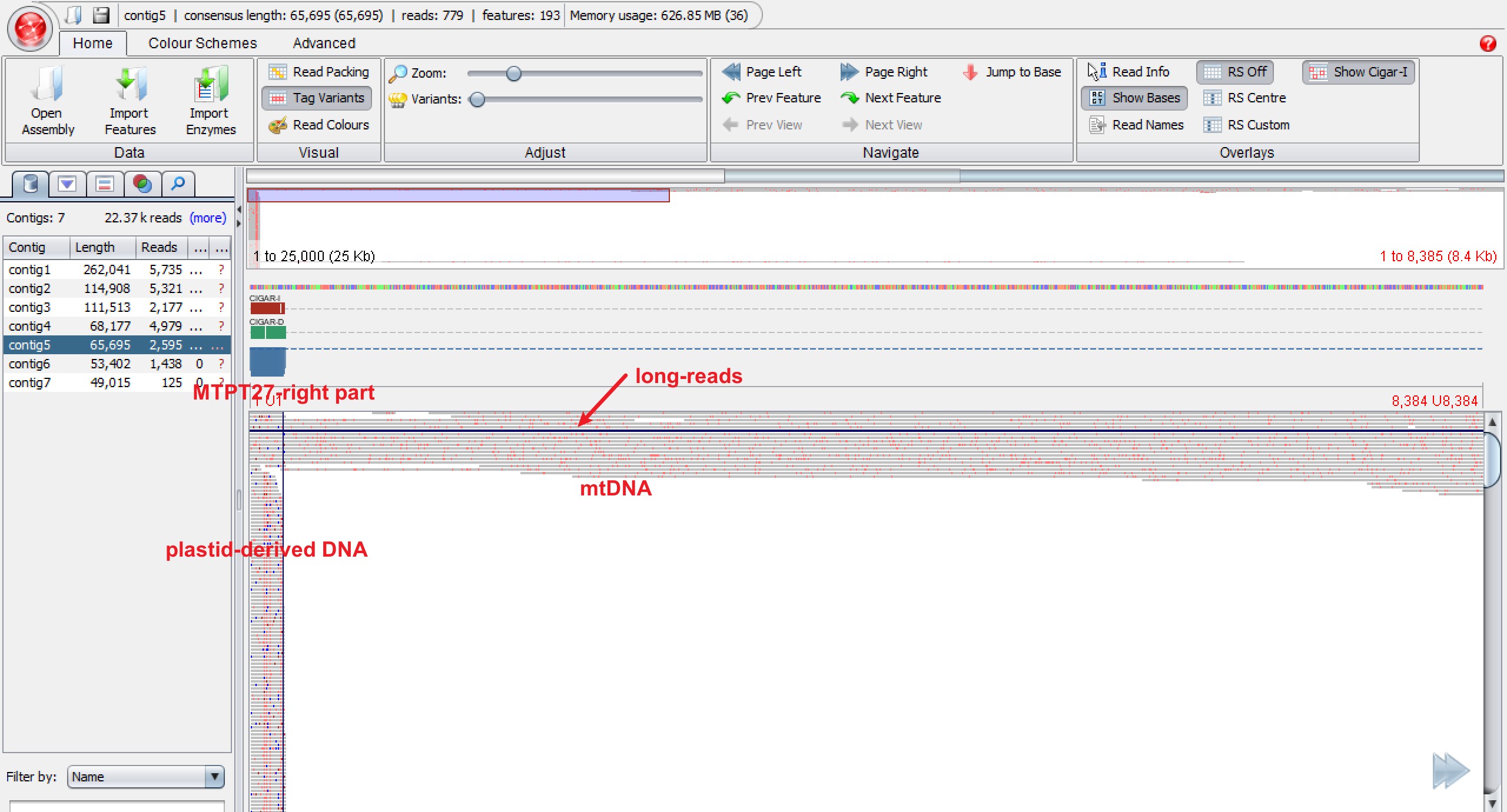

Supplement: Supplementary file 1 [file DataSheet1.ZIP › Supplementary Data/Supplementary Data4/MTPT27-right.jpg]

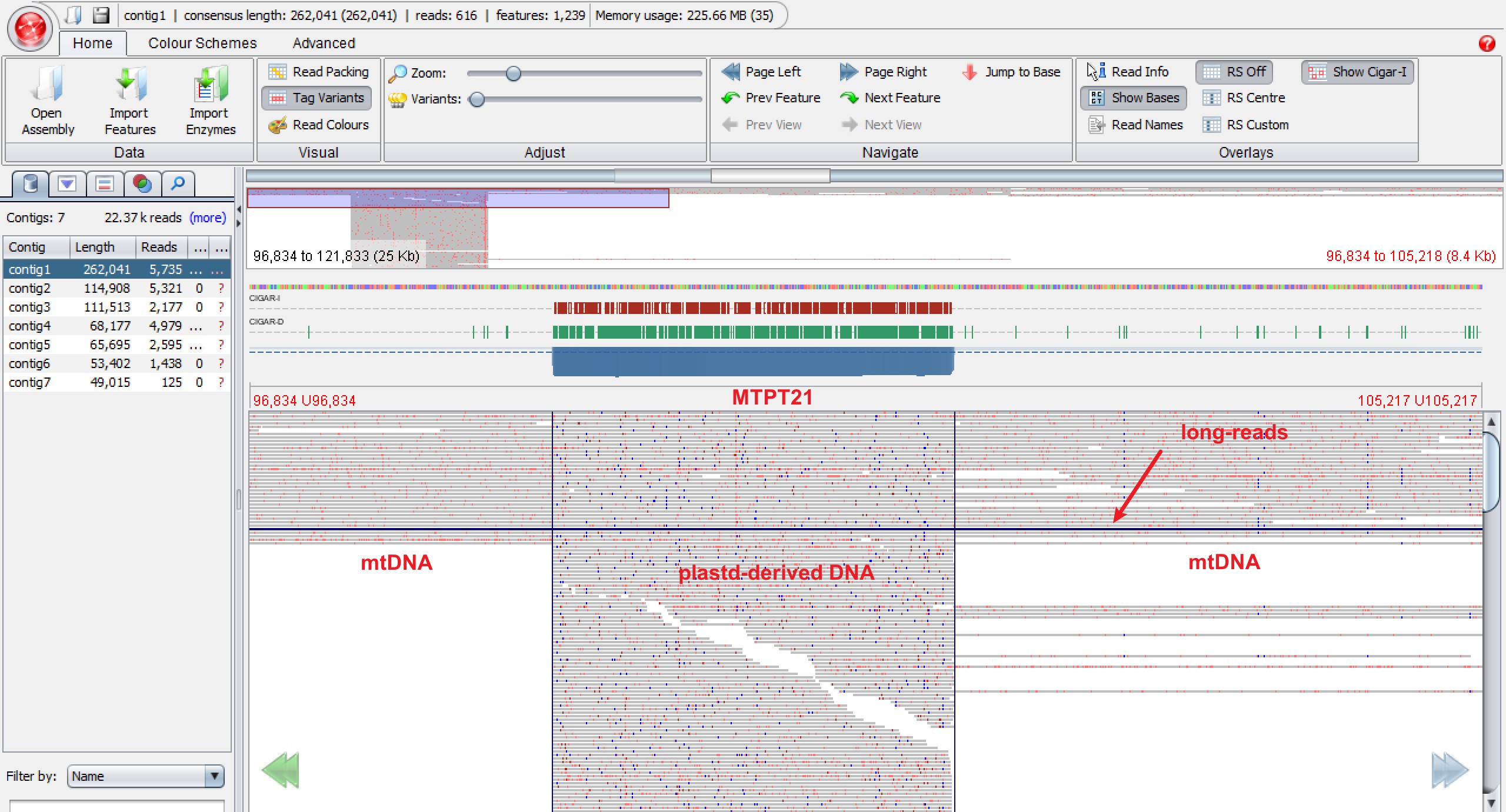

Supplement: Supplementary file 1 [file DataSheet1.ZIP › Supplementary Data/Supplementary Data4/MTPT21.jpg]

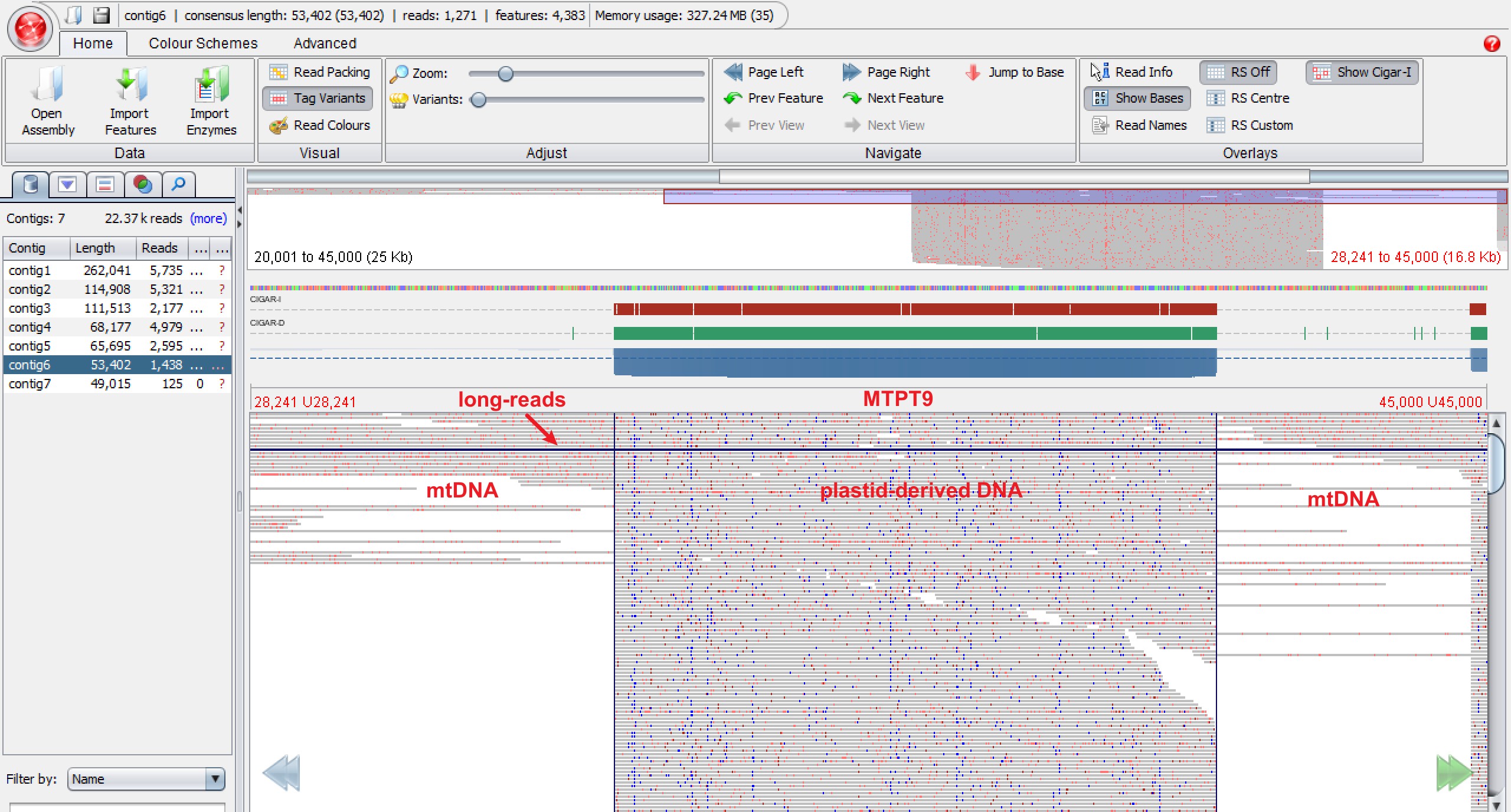

Supplement: Supplementary file 1 [file DataSheet1.ZIP › Supplementary Data/Supplementary Data4/MTPT9.jpg]
